# Facile Synthesis of Indium Sulfide/Flexible Electrospun Carbon Nanofiber for Enhanced Photocatalytic Efficiency and Its Application

**DOI:** 10.1155/2017/6513903

**Published:** 2017-12-20

**Authors:** Liu Han, Haohao Dong, Dong Mao, Baolv Hua, Qinyu Li, Dong Fang

**Affiliations:** ^1^School of Chemistry and Environmental Engineering, Yancheng Teachers University, Yancheng 224002, China; ^2^College of Chemical Engineering, Nanjing Tech University, Nanjing 210009, China

## Abstract

Heterojunction system has been proved as one of the best architectures for photocatalyst owing to extending specific surface area, expanding spectral response range, and increasing photoinduced charges generation, separation, and transmission, which can provide better light absorption range and higher reaction site. In this paper, Indium Sulfide/Flexible Electrospun Carbon Nanofiber (In_2_S_3_/CNF) heterogeneous systems were synthesized by a facile one-pot hydrothermal method. The results from characterizations of SEM, TEM, XRD, Raman, and UV-visible diffuse reflectance spectroscopy displayed that flower-like In_2_S_3_ was deposited on the hair-like CNF template, forming a one-dimensional nanofibrous network heterojunction photocatalyst. And the newly prepared In_2_S_3_/CNF photocatalysts exhibit greatly enhanced photocatalytic activity compared to pure In_2_S_3_. In addition, the formation mechanism of the one-dimensional heterojunction In_2_S_3_/CNF photocatalyst is discussed and a promising approach to degrade Rhodamine B (RB) in the photocatalytic process is processed.

## 1. Introduction

Nowadays, it is a huge challenge for people to deal with the organic pollutant in the energy crisis environment [1–3]. Certainly, photocatalytic as a novel solution has aroused great interest for people. It has been considered as one of the most effective ways for the solar energy conversion and the destruction of organic pollutant [4, 5]. Up to now, numerous experiments of the degradation of organic pollutants by using photocatalysts have been researched. However, the photocatalytic activity of pure photocatalyst is limited by its low efficiency of light absorption, difficult migration, and high recombination probability of photogenerated electron-hole pairs, and the development of photocatalytic technology is still limited for the photocatalyst [6–8]. Therefore, it is urgent and indispensable to find a novel photocatalyst to improve both the photochemical activity and the stability.

In_2_S_3_, as a typical III–VI group sulfide, is an n-type semiconductor with a band gap of 2.0–2.3 eV corresponding to visible light region which attracted intense interest for optical, photoconductive, and optoelectronic applications. Furthermore, In_2_S_3_ shows property of high photosensitivity and photoconductivity, stable chemical and physical characteristics, and low toxicity; it has great potential for visible-light-driven photodegradation of pollutants [9, 10]. Realistically, the narrow band gap and the rapid recombination of photogenerated electron-hole pairs causing poor quantum yield are similar to other visible light photocatalysts [11–14]. To meet the practical application requirements, it is urgent and important to enhance the photocatalytic efficiency of In_2_S_3_. Up to now, many attempts have been explored to improve the photocatalytic performance of In_2_S_3_, such as metal ions doping, coupling with other semiconductors, and carbon materials-based assemblies [15, 16].

As a viable alternative route to boost the efficiency of photocatalysts, CNF-based assemblies have aroused attention [17–19]. CNF is easily synthesized by electrospinning with a large theoretical specific surface area and high intrinsic electron mobility; it possesses physicochemical, superior electronic, mechanical character, and high absorption properties. In particular, compared with traditional carbon nanofibers obtained by other physical and chemical methods, the carbon nanofibers synthesized by electrospinning (CNF) have stronger electronic transport properties [20, 21]. Therefore, it is an ideal method to enhance photocatalytic activity by coupling In_2_S_3_ with CNF to construct In_2_S_3_/CNF.

**Scheme 1:**
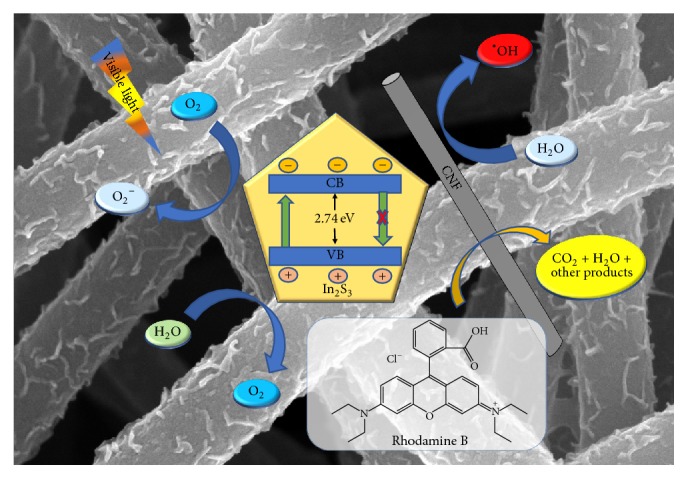
Postulated mechanism of the visible-light-induced photodegradation of RB with In_2_S_3_/CNF.

In this work, the CNF was fabricated by electrospinning technique, and In_2_S_3_/CNF composites were fabricated through a one-pot hydrothermal reaction as shown in Scheme 1. The photogenerated electrons on the conduction bands (CB) of In_2_S_3_ could easily be transferred to CNF for the positive synergetic effect, in brief, because the formation of interface junction can improve the optical absorption property and simultaneously facilitate the separation of photoinduced electron-hole pairs. In addition, the promising applications of In_2_S_3_/CNF composites have excellent performance for the degradation of organic pollutants. This study shows a reliable method to degrade organic pollutants.

## 2. Experimental Section

### 2.1. Materials

All the reagents were of analytical grade and were used as received without further purification. InCl_3_·5H_2_O, thioacetamide (TAA), and other chemicals were of analytical grade and purchased from Sinopharm Chemical Reagents Co., Ltd. Polyacrylonitrile (PAN) (*M*_*w*_ = 150,000 g mol^−1^) was purchased from Sigma-Aldrich.

### 2.2. Fabrication of CNF

According to previous reports [22], PAN nanofiber was synthesized from PAN by a modified electrospinning method. Firstly, 1 g PAN was dissolved completely in 9 mL* N*,*N*-dimethylformamide (DMF). Then, the mixture was transferred to 5 mL plastic syringe by two times for electrospinning (voltage: 20 kV, injection rate: 0.2 mm min^−1^). In order to obtain CNF, the PAN was carbonized at 500°C for 2 h under an inert atmosphere with a heating rate of 2 K min^−1^.

### 2.3. Fabrication of In_2_S_3_/CNF

In_2_S_3_/CNF with different In_2_S_3_ loadings was then prepared by a facile one-pot hydrothermal method. Briefly, a certain amount of InCl_3_·5H_2_O (351, 702, or 1053 mg) and thioacetamide (120 mg) was dissolved in ethyl alcohol (40 mL) under ultrasound conditions. The CNF (50 mg) was then immersed in the above solution, which was then transferred to a Teflon-lined autoclave and heated in a homogeneous reactor at 180°C for 12 h. According to this method, different weight ratios of the In_2_S_3_ to g-CNF samples were synthesized and labeled as In_2_S_3_/CNF-1, In_2_S_3_/CNF-2, and In_2_S_3_/CNF-4, respectively. By controlled trial, the In_2_S_3_ was fabricated by the same method.

### 2.4. Characterization

Scanning electron microscopy (SEM; Hitachi S-4800) coupled with X-ray energy dispersive spectroscopy (SEM-EDS) and transmission electron microscopy (TEM; Hitachi H600) were used to observe the morphology, structure, and size of the In_2_S_3_/CNF and its components. The effect of the In_2_S_3_ and CNF contents of In_2_S_3_/CNF on its structural properties were investigated by X-ray photoelectron spectroscopy (XPS; Axis Ultra HAS), Raman (Raman; Axis Ultra HAS), and X-ray diffraction (XRD; X' Pert-Pro MPD). The optical properties and the dye concentration were determined by UV-visible diffuse reflectance spectroscopy (UV-vis DRS, Shimadzu UV-3600).

### 2.5. Photocatalytic Activity Measurements

The photocatalytic activities of samples were evaluated by measuring the photodegradation of Rhodamine B (RB) under visible light. In a typical measurement, 40 mg photocatalysts were suspended in 100 mL of 50 ppm aqueous solution of RB. The solution was stirred in the dark for 30 min to obtain a good dispersion and to reach the adsorption–desorption equilibrium between the organic molecules and the catalysts surface [23]. Then the suspension was illuminated with a 250 W xenon lamp. The concentration change of RB was monitored by measuring the UV-vis absorption of the suspensions at regular intervals (take samples every 10 minutes). The suspension was filtered to remove the photocatalysts before measurement. The concentrations of RB in the reacting solutions were analyzed at *λ* = 554 nm [24]. The photocatalytic activity was analyzed by the time profiles of *C*/*C*_0_, where *C* is the concentration of RB at the irradiation time *t* and *C*_0_ is the concentration in the absorption equilibrium of the photocatalysts before irradiation, respectively. The normalized temporal concentration changes (*C*/*C*_0_) of RB are proportional to the normalized maximum absorbance (*A*/*A*_0_), which can be derived from the change in the RB absorption profile at a given time interval [25].

## 3. Results and Discussion

**Figure 1:**
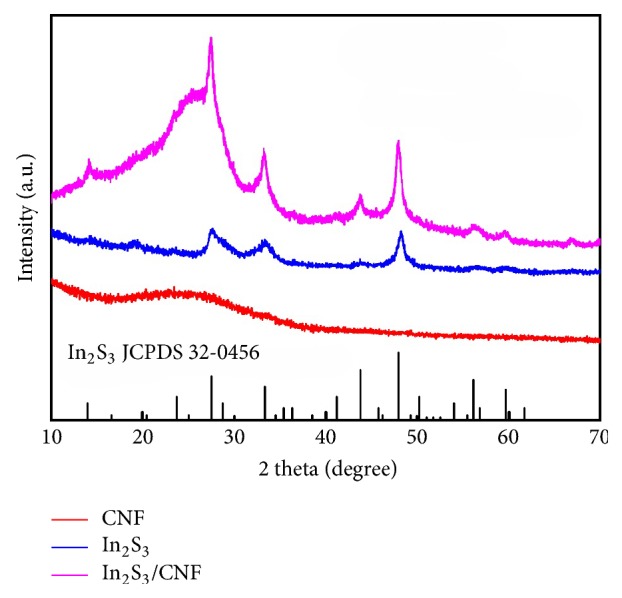
XRD patterns of In_2_S_3_, CNF, and In_2_S_3_/CNF-2.

The X-ray diffraction (XRD) patterns of pure CNF, In_2_S_3_, and In_2_S_3_ are shown in Figure 1. All of the diffraction peaks can be indexed to that of In_2_S_3_ with a cubic phase structure (JCPDS, number 32-0456). Peaks at 2*θ* of 14, 27, 33, 44, 48, 56, and 60° in the XRD patterns of In_2_S_3_/CNF-2 and In_2_S_3_ correspond to the (111), (311), (400), (511), (533), and (444) planes of In_2_S_3_, respectively. The XRD patterns of the In_2_S_3_/CNF-2 heterostructures show all the diffraction peaks assigned to hexagonal In_2_S_3_ except the peak at 25° which corresponds to (130) plane of orthorhombic CNF, indicating the existence of In_2_S_3_ and CNF in the In_2_S_3_/CNF-2 heterostructures. Moreover, the intensities of the corresponding diffraction peaks of In_2_S_3_ strengthened gradually along with the addition of the CNF in the In_2_S_3_/CNF-2 composites; the formation of heterostructures can be demonstrated.

**Figure 2:**
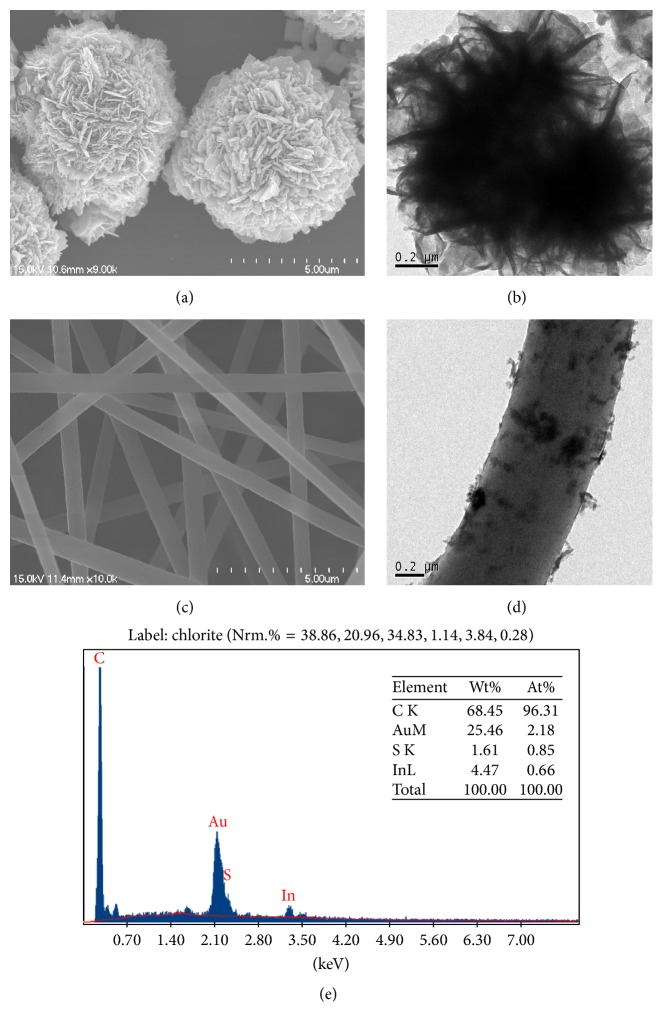
SEM images of In_2_S_3_ and CNF ((a) and (c)), TEM images of In_2_S_3_ and In_2_S_3_/CNF-2 ((b) and (d)), and EDX pattern of In_2_S_3_/CNF-2 (e).

The morphology of the In_2_S_3_ and In_2_S_3_/CNF-2 was analyzed by SEM and TEM. The flower-like In_2_S_3_ with an average diameter of 5 um possesses porous structures due to the aggregation of a certain amount of nanosheets (Figure 2(a)). The TEM image of Figure 2(b) further confirms the result. The SEM image of electrospun CNF is shown in Figure 2(c), which shows that the average diameter is about 300 nm and there is no defect in a smooth surface. As shown in TEM images (Figure 2(d)), it is clear that the surface of In_2_S_3_/CNF-2 is uniformly covered by the ultrathin In_2_S_3_ nanosheets after hydrothermal treatment. Further, there is no aggregation found in the surface of In_2_S_3_/CNF-2 composites.

The EDX spectrum shown in Figure 2(e) reveals the presence of In and S elements in a mass fraction ratio of 4.47% : 1.61%, which is close to the expected stoichiometry for In_2_S_3_ (Au signal is from FTO substrate).

**Figure 3:**
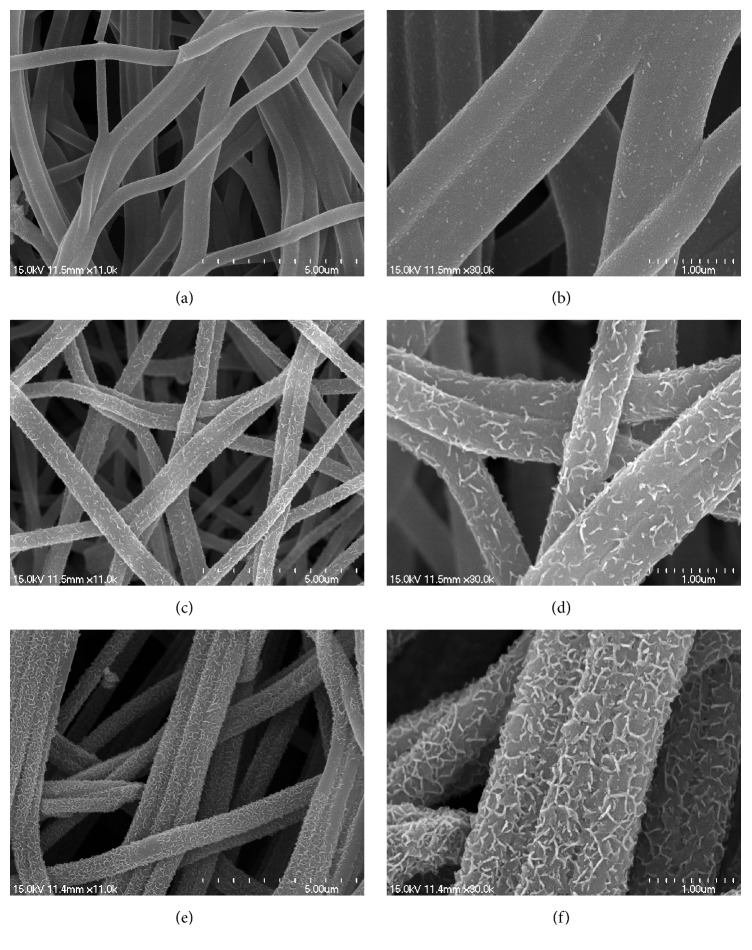
Low- (a, c, e) and high- (b, d, f) magnification SEM images of low concentration of In_2_S_3_ loaded on CNF ((a) and (b)), moderate concentration of In_2_S_3_ loaded on CNF ((c) and (d)), and high concentration of In_2_S_3_ loaded on CNF ((e) and (f)).

Figure 3 shows that the different concentration of In_2_S_3_ deposited on the surface of CNF nanofibers. A small amount of nanoplate-like In_2_S_3_ was found on the smooth surface of CNF nanofibers, which correspond to low concentration. As the concentration increases (Figures 3(c) and 3(d)), In_2_S_3_ nanosheets with curled shapes grow vertically on the nanofiber surface and with a uniform distribution. In addition, the surface of nanofiber also turns from smooth to rough. As shown in Figures 3(e) and 3(f), serious aggregation occurred and thick layer In_2_S_3_ nanosheets were observed after further increasing the In_2_S_3_ concentration. The rapid nucleation of In_2_S_3_ at high concentration can be demonstrated.

**Figure 4:**
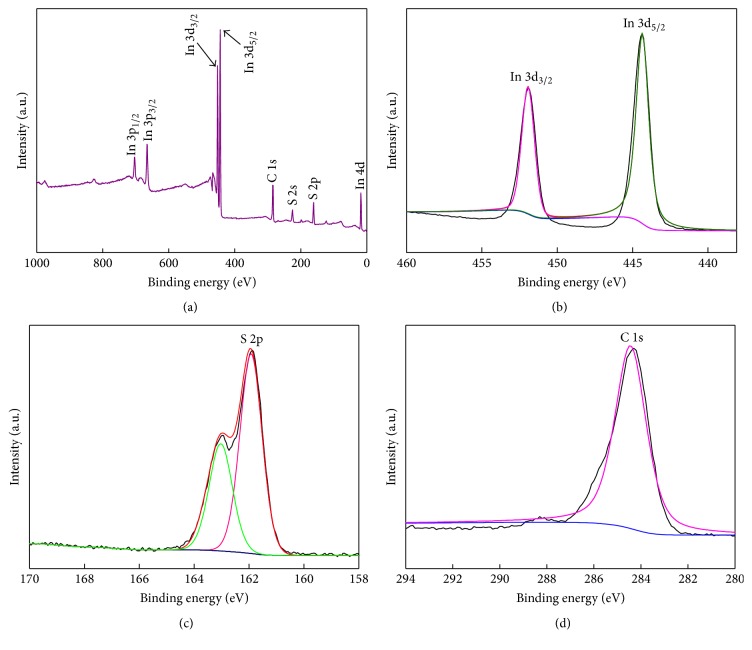
XPS spectra of the In_2_S_3_/CNF-2: survey spectrum (a), In 3d (b), S 2p (c), and C 1s (d).

XPS measurements were carried out to testify the chemical composition and chemical states of elements in In_2_S_3_/CNF-2 heterostructure photocatalyst [26]. The full-scale XPS spectrum for In_2_S_3_/CNF-2 sample is shown in Figure 4(a), in which the In, S, and C elements could be detected and no other impurities were observed. Figures 4(b), 4(c), and 4(d) show the high-resolution XPS spectra for In_2_S_3_/CNF sample. The XPS peaks (Figure 4(b)) at 444.1 and 452.7 eV correspond to the In3d_5/2_ and In3d_3/2_ states [27], respectively. The peak at 161.9 eV in Figure 4(c) corresponds to the S2p_3/2_ state of S_2_^2−^ moieties. The peak at 284.8 eV in Figure 4(d) corresponds to the C1s state. The above XPS results confirm that the composites are composed of In_2_S_3_ and CNF.

**Figure 5:**
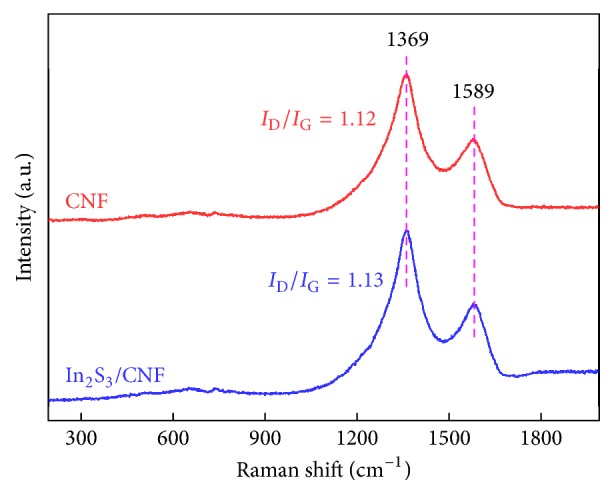
Raman spectra of CNF and In_2_S_3_/CNF-2.

Raman analysis was explored to confirm the presence of CNF and In_2_S_3_ in In_2_S_3_/CNF-2 sample (Figure 5). D-peak (D band) represents the defects of C atomic lattice, and G-peak (G band) represents the expansion vibration of the surface of C atom sp^2^ hybridization. And the representative Raman spectrum in a range of Raman shift from 100 to 2000 cm^−1^ of the CNF shows mainly two peaks centered around 1369 cm^−1^ (D band) and 1590 cm^−1^ (G band) for CNF. Furthermore, the degree of fibrosis can be measured by the intensity ratio of the G to D band (*I*_G_/*I*_D_) [28–30], where *I*_G_/*I*_D_ is the intensity ratio of D-peak and G-peak. A slight increase in the *I*_G_/*I*_D_ ratio is observed in the spectrum of In_2_S_3_/CNF-2 composites, the D/G integral intensity ratio (*I*_D_/*I*_G_) for CNF in the In_2_S_3_/CNF-2 sample (1.13) is slightly higher than that of CNF (1.12), it is indicated that a certain amount of In_2_S_3_ deposited on the surface of CNF during the chemical reduction process, and the conjugated CNF network was reestablished [31]. The two peaks for D and G band of the composite no shift appears, indicating that only a small amount of In_2_S_3_ deposited on the surface of CNF.

**Figure 6:**
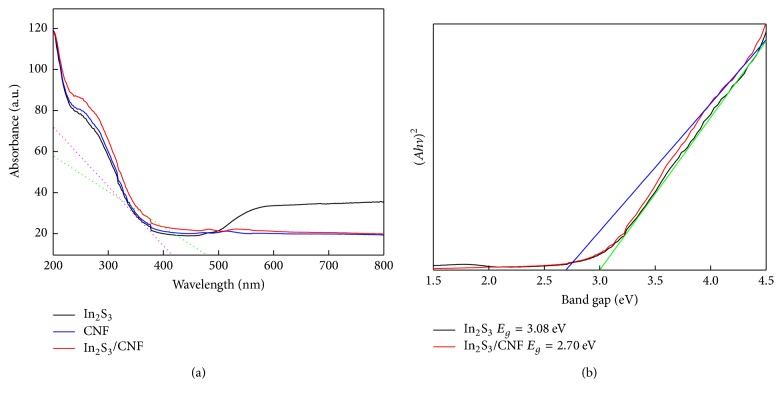
UV-vis diffuse reflectance spectrum of In_2_S_3_, CNF, and In_2_S_3_/CNF-2 (a) and the direct band gap determination of In_2_S_3_ and In_2_S_3_/CNF-2 (b). The tangent at this point corresponds to the smallest absorption wavelength.

The optical properties of the three samples were detected by UV-vis DRS absorption spectroscopy (Figure 6). Obviously, CNF shows the best performance and its absorption peaks appear in the visible light and UV light regions. It should be noted that In_2_S_3_/CNF-2 with the addition of CNF showed an increased photocatalytic performance compared to In_2_S_3_ (Figure 6(a)). The band gap energy (*E*_*g*_) of samples was calculated by Tauc's equation [32, 33] and the result was shown in Figure 6(b); the *E*_*g*_ values of In_2_S_3_ and In_2_S_3_/CNF-2 in Figure 6(b) are approximately 2.70 and 3.08 eV. The band gap of In_2_S_3_/CNF-2 was higher than In_2_S_3_, which is close to the value of In_2_S_3_ and In_2_S_3_/CNF reported in other literatures [10, 34]. Thus, it is indicated that the as-prepared In_2_S_3_/CNF-2 heterojunction structures have the appropriate *E*_*g*_ for photodegradation of organic pollutants under visible light irradiation.

**Figure 7:**
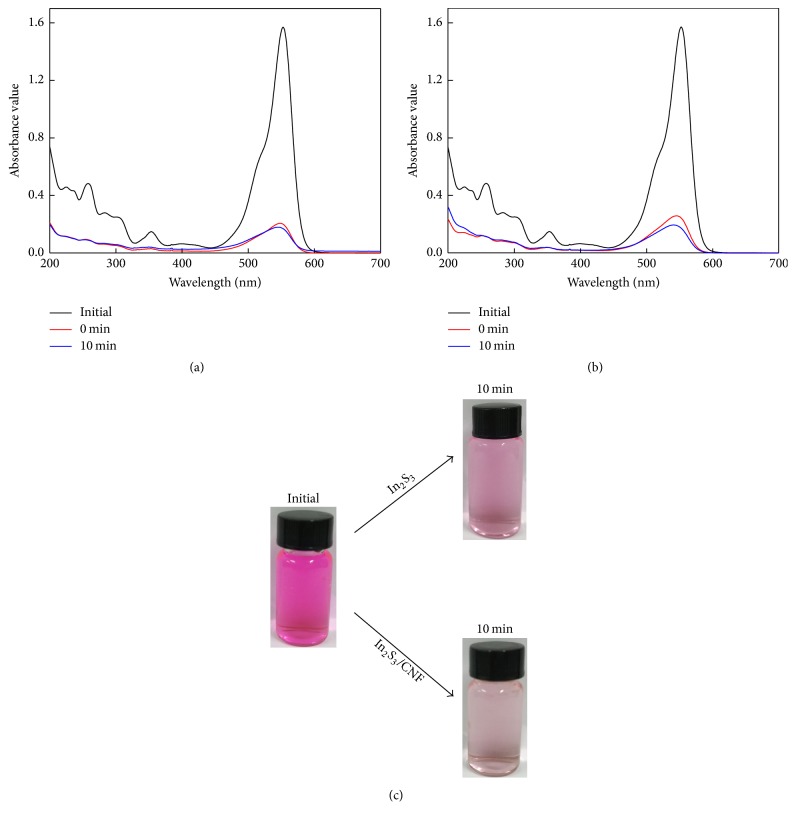
Removal efficiency of 50 mg/L RB solution during 10 min of irradiation with In_2_S_3_ (a) and In_2_S_3_/CNF-2 (b). The changes of solution color before and after degradation with different photocatalysts under visible light irradiation (c).

In order to detect the ability of photodegradation, different photocatalysts were used to photodegrade organic pollutant under visible light irradiation, then the samples of products were analyzed. The results are shown in Figures 7(a) and 7(b); the UV-vis absorption spectra in Figure 7 show the characteristic absorptions of RB at 570 and 580 nm. Owing to the strong absorption ability of CNF, a certain amount of RB was attached to the CNF before irradiation. Furthermore, when the dissociation and adsorption reach equilibrium, the concentration change of Rhodamine B, which is degraded by In_2_S_3_ and In_2_S_3_/CNF-2, is the same. The concentration of RB does not significantly change after irradiation as shown in Figure 7(a). The change in the concentration of RB in Figure 7(b) is significantly greater than Figure 7(a), which can be further confirmed through the change of solution color before and after degradation with different photocatalysts.

**Figure 8:**
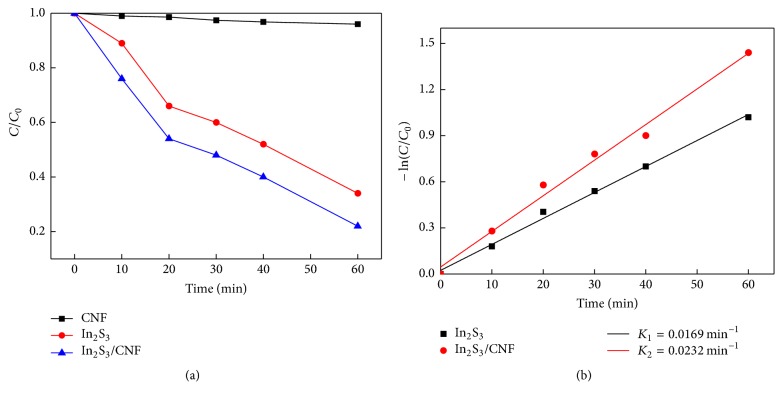
Degradation curve of RB over different photocatalysts under visible light (a). Kinetic linear simulation curves of RB degradation over the different photocatalysts under visible light irradiation (b).

The degradation curves of RB on pure In_2_S_3_, CNF, and In_2_S_3_/CNF-2 composites were shown in Figure 8(a). Obviously, the concentration of CNF almost has no change, indicating that pure CNF has no photocatalytic activity under visible light irradiation. The In_2_S_3_/CNF-2 composites have a better photocatalyic efficiency (78.2%) for RB after visible light irradiation for 60 min than that of pure In_2_S_3_. It is concluded that In_2_S_3_/CNF-2 composites exhibited much higher photocatalytic efficiency compared with the pure In_2_S_3_. For a better comparison of the photocatalytic efficiency of In_2_S_3_/CNF-2 and pure In_2_S_3_, the kinetic analysis of degradation of RB was explored to confirm it. The above degradation reactions followed a Langmuir-Hinshelwood apparent first-order kinetics model [32, 35] when the initial concentrations of the reactants are less than 100 ppm. The Langmuir-Hinshelwood apparent first-order kinetics model is described below:

(1) $$\begin{array}{c} -\ln\left(\;\frac{C_{0}}{C}\;\right)=K_{app}t, \end{array}$$

where *K*_app_ is the apparent first-order rate constant (min^−1^). The determined *K*_app_ values for degradation of RB with different catalysts are presented in Figure 8(b). It is clear that the as-prepared In_2_S_3_/CNF-2 composites show the highest reaction rate among the two catalysts with *K*_app_ = 0.0232 min^−1^, while *K*_app_ = 0.0169 min^−1^ for pure In_2_S_3_. The photocatalytic reactivity order is well consistent with the activity studies above.

It is reasonable to presume that the photogenerated electrons (e^−^) transfer from In_2_S_3_ to CNF in the In_2_S_3_/CNF system under visible light irradiation. Therefore, the photogenerated electrons first transfer to CNF and then are trapped by O_2_ and H_2_O at the surface of photocatalyst or solution to form the active species such as O_2_^−^. These active species could help the degradation of RB dye. At the same time, the photogenerated holes (h^+^) could react with H_2_O to form ^•^OH, hydrogen ions (H^+^), and then oxidize RB dye directly [12]. The complete photodegradation process can be summarized by the following reaction steps:

(2) In2S3+hν⟶In2S3e−+h+e−CB,In2S3+O2⟶O2−h+VB,CNF+H2O⟶H++OH•RB+O2−+h++OH•⟶several steps⟶other products+CO2+H2O

## 4. Conclusion

In summary, an effective method of preparing In_2_S_3_/CNF photocatalysts was described in this paper. The incorporation of CNF serving as electron collectors realizes a more effective separation of photogenerated electron-hole pairs and greatly boosts the photocatalytic activity of the products compared with the pure In_2_S_3_. The In_2_S_3_/CNF-2 composites show strong adsorption ability towards the RB, they can degrade 50 ppm of RB in 60 minutes under visible lights, and the excellent degradation RB activities of In_2_S_3_/CNF are mainly attributed to the large amount of effectively reactive species like h^+^ and O_2_^−^. Overall, this study provides a new option to construct the semiconductor/CNF composites with high photocatalytic activity, environmental remediation, and energy conversion.
